# Virus Infections and Sudden Death in Infancy: The Role of Interferon-γ

**DOI:** 10.3389/fimmu.2015.00107

**Published:** 2015-03-11

**Authors:** Sophia M. Moscovis, Ann E. Gordon, Osama M. Al Madani, Maree Gleeson, Rodney J. Scott, Sharron T. Hall, Christine Burns, Caroline Blackwell

**Affiliations:** ^1^School of Biomedical Sciences, Faculty of Health and Medicine, University of Newcastle and Hunter Medical Research Institute, Newcastle, NSW, Australia; ^2^Hunter Medical Research Institute, New Lambton, NSW, Australia; ^3^Medical Microbiology, University of Edinburgh, Edinburgh, UK; ^4^Hunter Area Pathology Service Genetics, John Hunter Hospital, New Lambton, NSW, Australia; ^5^Hunter Area Pathology Service Immunology, John Hunter Hospital, New Lambton, NSW, Australia

**Keywords:** sudden infant death syndrome, IFN-γ, ethnicity, cigarette smoke, cytokines

## Abstract

Respiratory infections have been implicated in sudden infant death syndrome (SIDS). As interferon-γ (IFN-γ) is a major response to virus infection, we examined (1) the frequency of single nucleotide polymorphism (SNP), *IFNG* T + 874A, in SIDS infants, their parents, and ethnic groups with different incidences of SIDS; (2) model systems with a monocytic cell line (THP-1) and human peripheral blood monocytes (PBMC) for effects of levels of IFN-γ on inflammatory responses to bacterial antigens identified in SIDS; (3) interactions between genetic and environmental factors on IFN-γ responses. *IFNG* T + 874A genotypes were determined for SIDS infants from three countries; families who had a SIDS death; populations with high (Indigenous Australian), medium (Caucasian), and low (Bangladeshi) SIDS incidences. The effect of IFN-γ on cytokine responses to endotoxin was examined in model systems with THP-1 cells and human PBMC. The IFN-γ responses to endotoxin and toxic shock syndrome toxin (TSST-1) were assessed in relation to genotype, gender, and reported smoking. There was a marginal association with *IFNG* T + 874A genotype and SIDS (*p* = 0.06). Indigenous Australians had significantly higher proportions of the *IFNG* T + 874A SNP (TT) associated with high responses of IFN-γ. THP-1 cells showed a dose dependent effect of IFN-γ on cytokine responses to endotoxin. For PBMC, IFN-γ enhanced interleukin (IL)-1β, IL-6, and tumor necrosis factor-α responses but reduced IL-8 and IL-10 responses. Active smoking had a suppressive effect on baseline levels of IFN-γ. There was no effect of gender or genotype on IFN-γ responses to bacterial antigens tested; however, significant differences were observed between genotypes in relation to smoking. The results indicate virus infections contribute to dysregulation of cytokine responses to bacterial antigens and studies on physiological effects of genetic factors must include controls for recent or concurrent infection and exposure to cigarette smoke.

## Introduction

The peak in sudden infant death syndrome (SIDS) occurs during the developmental period in which infants have low levels of specific antibody protection, either maternal or actively acquired immunity. They are dependent on their innate responses to deal with new infectious agents they encounter in their environment. Infection and inflammatory responses have been implicated in many of these deaths and we have examined the hypothesis that some SIDS deaths occur as a result of dysregulation of inflammatory responses which can affect physiological systems suggested to be involved in triggering these deaths (1).

There is a growing body of evidence that infection (1–3) and the inflammatory response to these infections (1, 4, 5) play a role in both SIDS and sudden unexpected death in infancy (SUDI). Inflammatory changes, particularly in the respiratory tract, are common findings in SIDS. It has been suggested that these findings reflect recent infections, symptoms of which have been noted in the 2 weeks prior to death for over 40% of SIDS infants (6, 7). Mild respiratory infections have been noted prior to death, but no single virus has been implicated (8). In infants, virus infections have been demonstrated to enhance the number and variety of bacterial species in the nasopharynx (9), particularly among infants sleeping in the prone position. While specific virus infections were not identified among SIDS infants, it was noted that there was a significantly higher proportion of coliform organisms in the respiratory tract of many (10). Both *Staphylococcus aureus* and *Escherichia coli* were identified in a significantly higher proportion of SUDI infants than infants who died of known causes (2, 3).

In animal models, induction of pro-inflammatory cytokines such as interferon-γ (IFN-γ) contribute to the severity of a host’s responses to either infectious agents or their products, and this can be greatly enhanced by a co-existing virus infection (11–14). Toxigenic bacteria or their toxins have been implicated in the etiology of SIDS (15–20). Priming with an asymptomatic virus infection can significantly reduce the concentration of bacterial toxins needed to induce death.

There have been reports that levels of IFN-γ responses differ between the single nucleotide polymorphisms (SNPs) genotypes (21, 22). We have demonstrated in previous studies that these can vary among different ethnic groups (23) and that there are interactions between genotypes and exposure to environmental agents such as cigarette smoke (24). As virus infections and exposure to cigarette smoke are both significant risk factors for SIDS, our study tested the following hypotheses: (1) the single nucleotide polymorphism *IFNG* T + 874A genotype associated with higher IFN-γ responses might be over-represented among SIDS infants or ethnic groups in which there is a higher incidence of SIDS; (2) as a surrogate for virus infection, exposure to IFN-γ would significantly alter cytokine responses from human leukocytes to bacterial antigens identified in SIDS infants; (3) cigarette smoking might affect IFN-γ responses to bacterial toxins *in vitro*, as noted previously for other cytokines (24, 25).

## Subjects and Methods

Approval for the study was obtained from the Lothian Health Ethics Committee (UK), Hunter Area Research Ethics Committee (Australia), and the University of Newcastle Human Research Ethics Committee (Australia).

### Assessment of *IFNG* T + 874A SNP

Buccal epithelial cells were collected from Caucasian parents of SIDS infants from Britain (*n * = 34) and Australia (*n * = 60), and their matched controls with no family history of SIDS (Britain *n * = 59, Australia *n * = 55).

Paraffin-fixed samples of tissue from SIDS infants were obtained from Australia (*n * = 17), Hungary (*n * = 21), and Germany (*n * = 47). Stored frozen whole blood samples from Indigenous Australians (*n * = 123) and buccal epithelial cells from Bangladeshis (*n * = 32) were used as DNA sources for comparisons between ethnic groups. The methods for extraction of DNA from the samples have been described previously (24–26).

To genotype *IFNG* T + 874A (rs2430561) a custom made allelic discrimination polymerase chain reaction (PCR) assay was manufactured (PE Applied Biosystems). Primers: 5′ GCT GTC ATA ATA ATA TTC AGA CAT TCA CAA TTG AT 3′; 5′ TGC GAG TGT GTG TGT GTG T 3′ and probes: 5′ CAC AAA ATC AAA TCT CAC ACA C 3′; 5′ ACA AAA TCA AAT CAC ACA CAC 3′ were provided in a 40× assay mix.

Each PCR reaction contained 10 ng of sample DNA, 1× Assay mix, and 1× TaqMan Universal PCR Master Mix (PE Applied Biosystems) made up to a final volume of 5 μl with sterilized MilliQ water. PCR was performed using the ABI PRISM 7900HT sequence detection system (PE Applied Biosystems) at the following thermal cycling conditions: 50°C for 2 min; 95°C for 10 min; 92°C for 15 s; and 60°C for 1 min, for 40 cycles.

Data were analyzed using the statistical software package Statistics/Data Analysis™(STATA) Version 8.0 (Stata Corporation, College Station, TX, USA). The Chi-square (χ^2^) test or Fisher’s exact test, if appropriate, was used to assess the distribution of *IFNG* T + 874A in SIDS infants, parents of SIDS infants, and between ethnic groups. Deviation of *IFNG* T + 874A genotype distribution from Hardy–Weinberg equilibrium (HWE) was assessed using the χ^2^ test.

### Assessment of the effects of IFN-γ on responses to bacterial antigens

The THP-1 human monocytic cell line used in previous studies of inflammatory responses to bacterial endotoxin (27) was used in preliminary studies to determine concentrations of components to be used in the model system (28).

Collection, isolation, and storage of human peripheral blood monocytic cells (PBMC) have been described previously (29). Buffy coats from 14 male (*n * = 14) and 14 female (*n * = 14) donors, aged 20–55 years, were purchased from the Australian Red Cross Blood Service (ARCBS) (Sydney, NSW, Australia). Ethical permission was obtained from University of Newcastle Human Research Ethics Committee (H-229-0606) and ARCBS Ethics Committee (07-11NSW-07) for the purchase and use of human buffy coats for the purposes of the study. PBMC were collected from each donor for *in vitro* cytokine stimulation assays and plasma was collected for the assessment of cotinine for evidence of exposure to cigarette smoke, a confounding variable for altered cytokine responses. Donors with detectable levels of cotinine were excluded from the analysis. Only ARCBS donor samples that were cleared for infectious agents were received. Buffy coats were processed within 24 h of collection.

Blood samples (10–20 ml) were collected from British parents of SIDS infants (*n* = 34) and control individuals (*n* = 59) who had no family history of SIDS. Leukocytes were isolated and stored as described previously (24–26).

### Analysis of exposure to cigarette smoke

As described previously (29), plasma from the samples from ARCBS donors were assessed for exposure to cigarette smoke by a semi-quantitative commercial competitive enzyme immunoassay (EIA) kit according to manufacturer’s instructions (OraSure Technologies Inc., Bethlehem, PA, USA). To prevent false negative classification of exposure to cigarette smoke, the qualitative cut-off of the assay was lowered from the recommended 25 to 10 ng ml^−1^. Donors with detectable levels of cotinine were excluded from stimulation assays.

### Stimulation assays

Conditions for experiments with the THP-1 cells have been described previously (28).

Peripheral blood monocytic cells from the ARCBS donors (*n * = 28) were assessed for *in vitro* cytokine responses to a common bacterial antigen, *E. coli* lipopolysaccharide (LPS) (50 ng ml^−1^). IFN-γ (10 ng ml^−1^) was used as a surrogate for virus infection. A water-soluble cigarette smoke extract (CSE) was used as a surrogate for exposure to cigarette smoke. The method has been described previously (29).

The leukocytes from British parents of SIDS infants were assessed for inflammatory responses to TSST-1 and LPS as described previously (24–26). All samples were coded and tested without knowing the smoking status of the donors. Leukocytes (1 × 10^6^ cells ml^−1^) were stimulated *in vitro* with 0.01, 1 μg ml^−1^ *E. coli* endotoxin or 0.5 μg ml^−1^ toxic shock syndrome toxin (TSST-1) (Sigma, Poole, Dorset, UK) for 24 h. Cell culture conditions have been described previously (24–26).

### Analysis of cytokine responses

Supernatants from the THP-1 and PBMC assays were measured for IL-1β, IL-6, tumor necrosis factor-α (TNF-α), IFN-γ, IL-8, and IL-10 using Bio-Rad 6-plex assays and the Luminex 200 analyzer. Cytokine concentrations (pg ml^−1^) were calculated from the standard curve using the Luminex 2.3 Software. Data were analyzed using STATA™ Version 10.0. Mean donor cytokine measurements were used for statistical analysis. The Wilcoxon matched-pairs signed ranks test was used to assess differences in cytokine responses between treatment groups. The significance level was set at *p* < 0.05.

Supernatants from assays with leukocytes from the British SIDS and control families were measured for IFN-γ levels using enzyme-linked immunosorbent assays (ELISA) kits (R&D Systems, Minneapolis, MN, USA) according to manufacturer’s instructions. Results were expressed as ng ml^−1^ derived from the standard curves obtained using a recombinant human IFN-γ standard (R&D Systems). The result for each group assessed was reported as the median value. Student’s *t*-test was used on log-transformed data to assess differences in IFN-γ responses of smokers and non-smokers and of males and females in relation to genotype. The significance level for all tests was set at *p * < 0.05.

## Results

### Analysis of genotypes of *IFNG* T + 874A

The distributions of *IFNG* T + 874A genotypes among the different groups assessed are summarized in Table 1. Distributions were in HWE for each of the groups assessed, except for the Australian SIDS infants.

**Table 1:** ***IFNG* T + 874A allele frequency distributions across populations**.

Ethnicity	Group		Allele frequency (%)			Sample size (*n*)	*p* Value (*p*)
			TT	TA	AA	
British	SIDS	Parents	12	56	32	34	0.53
British	Control	Parents	14	44	42	59	
Australian	SIDS	Parents	13	55	32	60	0.77
Australian	Control	Parents	18	51	31	55	
Australian	SIDS	Infants	25	19	56	16	0.06^a^
Hungarian	SIDS	Infants	13	50	38	8	0.89^b^ 0.39^c^
German	SIDS	Infants	22	47	31	45	0.12^d^
Combined	SIDS	Infants	22	41	38	69	0.52^e^
Bangladeshi	Control		13	50	38	32	0.93^f^
Indigenous Australian	Control		39	49	12	75	<0.01^g^ <0.01^h^
Caucasian	Control		16	47	37	114	

*^a^Australian SIDS infants vs. Australian control parents*.

*^b^Hungarian SIDS infants vs. German SIDS infants*.

*^c^Hungarian SIDS infants vs. Australian SIDS infants*.

*^d^German SIDS infants vs. Australian SIDS infants*.

*^e^Combined SIDS infants vs. Caucasian control*.

*^f^Bangladeshi control vs. Caucasian control*.

*^g^Indigenous Australian vs. Caucasian control*.

*^h^Indigenous Australian vs. Bangladeshi control*.

### Distribution of *IFNG* T + 874A among SIDS infants

There were no significant differences in the *IFNG* T + 874A distribution in SIDS infants from different countries (Table 1). The predominant genotype among Australian SIDS infants was AA (9/16, 56%); approximately half of Hungarian (4/8, 50%) and German (21/45, 47%) infants possessed the TA genotype.

The distribution of *IFNG* T + 874A in the Australian control population approached a significant difference from that observed for Australian SIDS infants (*p * = 0.06). The genotype distribution of the Australian SIDS infant group was inversely proportional to the distribution of the control population. There was a predominance of the TT (25%) and AA (56%) genotypes, and an under representation of the TA (19%) genotype. The Australian control population was similar to all other populations, with approximately half the population (51%) with the TA genotype. The under representation of the TA genotype in the Australian SIDS infant group caused the genotype distribution to be significantly different from that of the HWE (*p * = 0.02). No significant differences were detected between the distributions of *IFNG* T + 874A genotype for the combined SIDS infant group compared to the Caucasian controls (*p * = 0.52).

### Assessment of *IFNG* T + 874A among parents of SIDS infants

Differences in the distribution of *IFNG* T + 874A genotype among parents of SIDS infants were not statistically significant compared to their respective control populations (Table 1). Parents of SIDS infants recruited from Britain showed an increased proportion of individuals with the TA genotype compared to the matched British controls but this was not significant (*p * = 0.53). Parents of SIDS infants recruited from Australia had a similar distribution to their control population, with the majority of individuals possessing the TA genotype (*p * = 0.77).

### Distribution of *IFNG* T + 874A in different ethnic groups

As there were no differences between the distribution of *IFNG* T + 874A genotype for British and Australian control populations, these data were combined for further comparison with samples from ethnic groups with low incidences of SIDS (Bangladeshi) or high incidences (Indigenous Australian) compared with Caucasian populations. The majority of each population possessed the TA genotype. The distribution of *IFNG* T + 874A genotype was similar for the Caucasian and Bangladeshi populations; however, both were significantly different from the Indigenous Australian population (*p * < 0.01) (Table 1). The Aboriginal Australian population had a significant increase in the proportion of the individuals with the TT genotype compared to the Caucasian and Bangladeshi populations.

### Effects of IFN-γ and CSE on cytokine responses elicited by LPS from THP-1 cells

**Figure 1:**
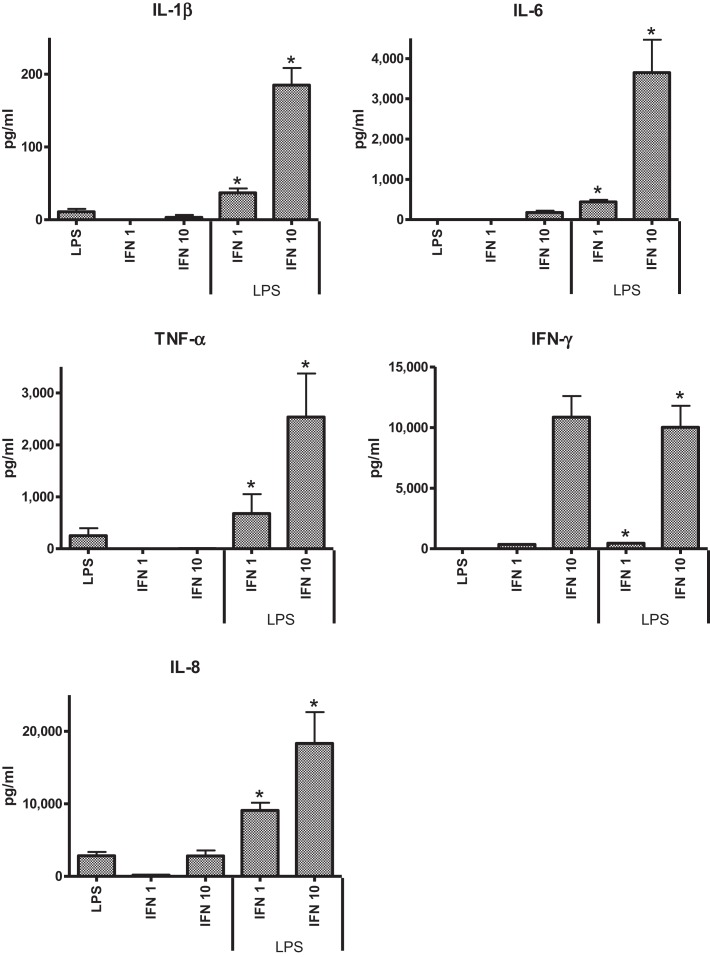
**Effects of IFN-γ, 1 ng ml^−1^ (IFN 1) or 10 ng ml^−1^ (IFN 10) on cytokine responses of THP-1 cells to LPS (50 ng ml^−1^)**.

In the preliminary experiments with THP-1 cells, two concentrations of IFN-γ were tested (1 and 10 ng ml^−1^) to assess the effect of dose on pro-inflammatory responses to LPS. TSST was not assessed in these preliminary studies as previous work indicated that THP-1 cells did not respond to the toxin. IL-6 was not elicited from THP-1 cells in the absence of IFN-γ. The higher concentration of IFN-γ induced significantly higher levels of IL-1β, IL-6, and TNF-α (Figure 1). The anti-inflammatory IL-10 was not detected under any of the conditions tested.

Because our previous studies indicated cigarette smoke exposure could significantly affect cytokine responses in the model systems tested (24), we assessed the effects of CSE alone or CSE and IFN-γ on responses to LPS. Pre-treatment of THP-1 cells with CSE resulted in decreased responses to LPS. Although the higher dose of CSE (0.001 cigarette ml^−1^) appeared to reduce IL-1β, TNF-α, and IFN-γ responses, the effect was not significant. Both low and high concentrations of CSE-enhanced IL-8 responses, but these were not significant. CSE exposure did not have a significant effect on responses induced with LPS from IFN-γ primed THP-1 cells.

### Effects of IFN-γ and CSE on cytokine responses from PBMC

**Figure 2:**
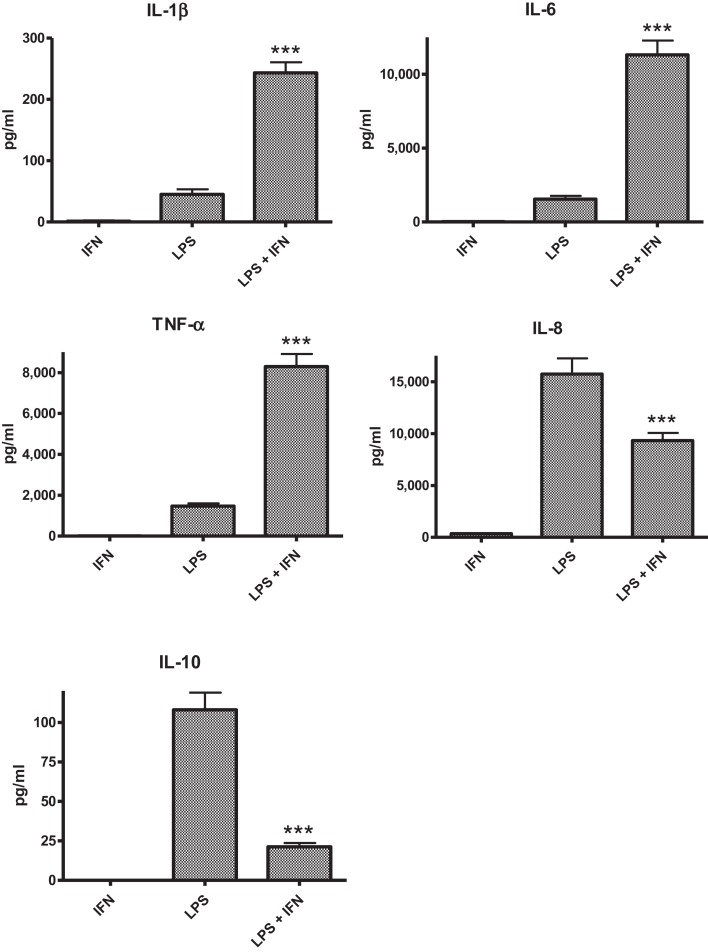
The effect of IFN-γ (10 ng ml^−1^) pre-treatment on LPS (50 ng ml^−1^) stimulation of IL-1β, IL-6, TNF-α, IL-8 and IL-10 from PBMC (*n * = 28) ***p < 0.0001.

**Figure 3:**
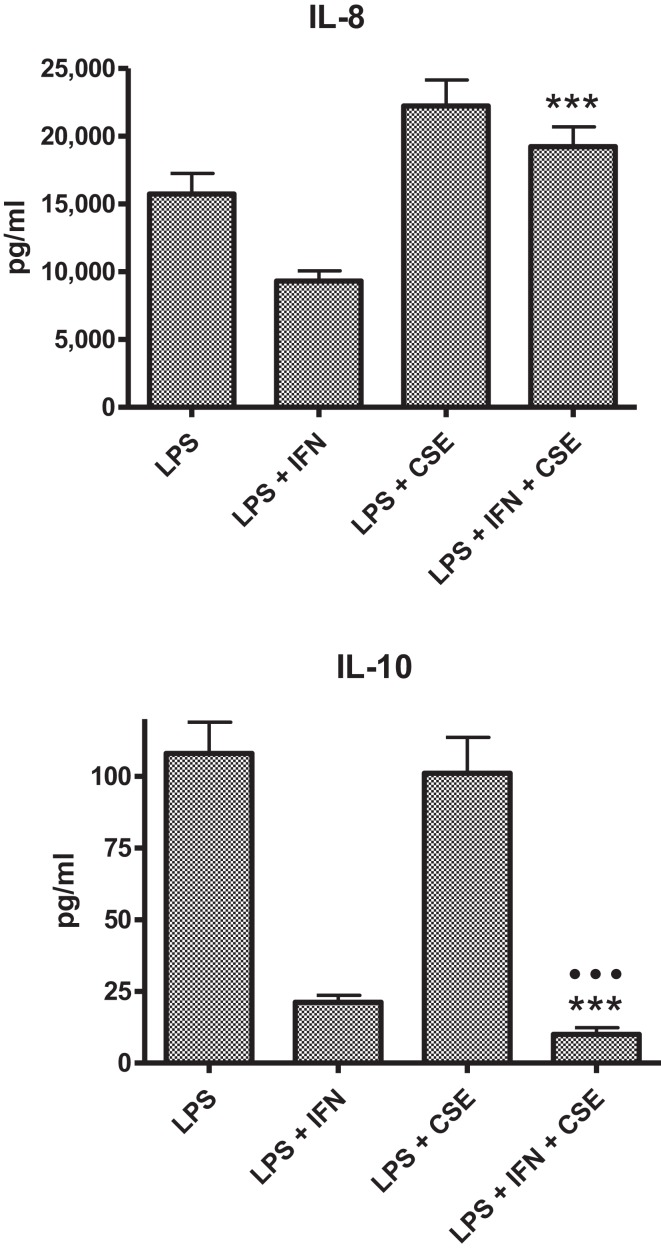
Combined effects of IFN-γ and CSE on IL-8 and IL-10 responses of PBMC to LPS (*n* = 28) ***p < 0.0001.

The PBMC from 28 donors were used to assess the effect of priming with 10 ng ml^−1^ IFN-γ on cytokine responses to LPS. A similar pattern of enhancement of pro-inflammatory responses elicited by the IFN-γ-primed cells was observed with PBMC from blood donors. CSE exposure reduced the effects of IFN-γ priming on LPS stimulation of pro-inflammatory cytokines IL-1β, IL-6, and TNF-α. In contrast to results with THP-1 cells, IFN-γ priming significantly reduced IL-8 responses and the anti-inflammatory IL-10 responses of PBMC to LPS (Figure 2). LPS stimulated cells pre-treated with both IFN-γ and CSE had the lowest responses for both IL-8 and IL-10 (Figure 3).

### Effect of smoking, genotype, and gender on IFN-γ responses to LPS or TSST-1

The assays with PBMC from SIDS parents and unrelated individuals were used to assess the effects of smoking and genotype on IFN-γ responses to LPS or TSST-1. The results are summarized in Table 2 and described below.

**Table 2:** **Median IFN-γ levels of 1 × 10^6^ cells/ml leukocytes stimulated *in vitro* with TSST and endotoxins (LPS), assessed by *IFNG* T + 874A genotype and smoking status**.

Smoking status	*IFNG* + 874A	Gender	Median IFN-γ response (ng/ml) to toxin (μg/ml)				Sample size (*n*)
			Baseline	TSST-1 1	LPS 1	LPS 0.01	
Smoker	All	All	1	1,542	467	55	45
Non-smoker			4	1,074	559	88	74
All	TT	All	1	895	821	51	15
	TA		1	1,357	613	83	48
	AA		5	1,452	477	90	41
All	All	Male	1	893	874	88	26
		Female	1	1,135	620	57	52
Smoker	TT	All	1	1,039	440	2	4
	TA		1	2,040	550	70	11
	AA		2	1,293	468	75	17
Non-smoker	TT		3	895	928	146	31
	TA		3	1,136	623	86	18
	AA		7	1,477	525	104	23
Smoker	All	Male	1	1,950	1,120	94	10
Non-smoker			3	567	490	88	16
Smoker		Female	1	927	490	48	19
Non-smoker			5	1,167	638	69	33
Smoker	TT	Male	1	2,857	821	3	1
	TA		1	2,121	2,260	432	4
	AA		1	1,822	359	16	3
Non-smoker	TT		1	215	812	158	2
	TA		1	647	406	83	9
	AA		386	550	570	421	2
Smoker	TT	Female	1	1,039	607	12	2
	TA		2	808	340	51	6
	AA		5	1,293	664	75	10
Non-smoker	TT		6	1,818	1,411	214	6
	TA		1	1,151	1,243	51	10
	AA		7	831	477	69	15

#### Smoking

Smoking had a small but significant suppressive effect on baseline IFN-γ levels (1 ng ml^−1^) compared to non-smokers (4.15 ng ml^−1^) (*p * < 0.01). This effect disappeared when cells were stimulated with 0.01 or 1 μg ml^−1^ LPS or 1 μg ml^−1^ TSST.

#### Genotype

When analyzed irrespective of smoking status, there were no significant differences between IFN-γ responses among *IFNG* T + 874A genotypes for either of the stimulants.

#### Gender

Overall, there were no differences in IFN-γ responses of cells from males compared with those from females. When smoking status was assessed in relation to gender, there was a significant difference in the IFN-γ response to 1 μg ml^−1^ TSST-1 for non-smokers: males (567 ng ml^−1^); females (1,167 ng ml^−1^) (*p * = 0.01). This trend was also observed for 1 μg ml^−1^ LPS; however, differences were not significant (*p* > 0.05).

### Interactions between genotype and smoking in relation to IFN-γ responses to LPS

The IFN-γ responses of cells from donors with the TT genotype to 0.01 μg ml^−1^ LPS was significantly decreased in smokers (1.85 ng ml^−1^) compared to non-smokers (145.8 ng ml^−1^) (*p * = 0.02). For non-smokers, the reverse pattern was observed; IFN-γ responses of TT donors were higher than those of the other genotypes but the differences were not significant. The IFN-γ responses to 0.01 μg ml^−1^ LPS from cells of smokers with the TT genotype (1.85 ng ml^−1^) was significantly lower compared to those from cells of smokers with the TA (69.9 ng ml^−1^) (*p * < 0.01) or AA genotypes (75.1 ng ml^−1^) (*p * = 0.01).

### Interactions between genotype and smoking in relation to IFN-γ responses to TSST

Smokers had lower baseline IFN-γ levels than non-smokers. For individuals with the AA genotype, smokers had significantly lower IFN-γ responses (1.85 ng ml^−1^) compared to non-smokers (6.6 ng ml^−1^) (*p * = 0.03).

Differences between genotypes were observed for smokers when cells were stimulated with 1 μg ml^−1^ TSST. Cells from smokers with the TA genotype (2,039 ng ml^−1^) had significantly higher IFN-γ responses than those with the TT (1,039 ng ml^−1^) (*p * = 0.03) or AA genotypes (1,292 ng ml^−1^) (*p* = 0.05) (Table 2).

When genotypes were assessed in relation to smoking status, there was a significant difference for the TA genotype. Cells from smokers had higher IFN-γ responses to 1 μg ml^−1^ TSST (2,039.7 ng ml^−1^) compared to non-smokers (1,136.1 ng ml^−1^) (*p * = 0.01).

## Discussion

We found no significant associations with the *IFNG* T + 874A SNP genotypes and SIDS. As found for the previous report on a British population (30), there was a marginal difference between the SIDS and local control groups (*p * = 0.06) in the distribution of the *IFNG* T + 874A genotype; however, the distribution of the genotypes between European and Australian SIDS infants was different. Among the European infants, the TA genotype was predominant; but among the Australian infants the AA genotype was predominant (56%).

It is not unusual for cytokine gene polymorphisms to differ between countries, particularly among disease cohorts. Differences between countries reinforce the need for local controls and caution exercised for interpretation of differences between disease and control groups from different ethnic backgrounds and countries.

Due to the under representation of the TA genotype, the genotype distribution of the Australian SIDS infant group was out of HWE. This was observed for two other SNPs previously assessed in this population; *IL1RN* T + 2018C and *IL10* G-1082A (24, 26). The decrease in the heterozygous genotype of *IFNG* T + 874A, *IL1RN* T + 2018C, and *IL10* G-1082A in the Australian SIDS infant population indicates that there is bias in the selection of the population. This might highlight the importance of these SNPs in the etiology of SIDS in Australia.

This study has again demonstrated the differences in distributions of cytokine gene polymorphisms among ethnic groups. There is an increased proportion of individuals with *IFNG* T + 874A TT genotype in the Indigenous Australian population compared to Caucasian and Bangladeshi populations. No Indigenous Australian SIDS infants were examined in the study.

In general, the effect of IFN-γ was similar for both THP-1 and PBMC models, enhancing pro-inflammatory responses IL-1β, IL-6, and TNF-α to stimulation with LPS. In contrast to results obtained with THP-1 cells, IFN-γ significantly reduced IL-8 responses of PBMC to LPS. Addition of CSE to the THP-1 cells primed with IFN-γ did not significantly alter the responses compared with priming cells with IFN-γ only. Addition of CSE to the PBMC primed with IFN-γ reduced the enhanced IL-1β, IL-6, and TNF-α responses to LPS. IFN-γ significantly reduced the CSE-enhanced IL-8 responses to LPS. Potentially, the most significant physiological effect was the reduction in IL-10 responses in the presence of both IFN-γ and CSE (Figure 3). If the effects of these two risk factors for SIDS demonstrated *in vitro* reflect the responses to infection *in vivo*, the combined effects of cigarette smoke and virus infection on reduction of IL-10 could result in significant dysregulation of pro-inflammatory responses. We have previously demonstrated a dose-dependent reduction in pro-inflammatory responses in our model system with increasing levels of IL-10 (24).

Genotype alone was not responsible for differences in IFN-γ responses. When variables were assessed independently, few differences were observed. Smoking had a small, but suppressive effect on baseline IFN-γ levels. There was no effect of gender or *IFNG* T + 874A genotype on IFN-γ responses.

When the effects of smoking status and gender on IFN-γ responses were assessed, the only difference between genders was among non-smokers; males had reduced IFN-γ response to TSST-1. Gender differences in cytokine responses have been previously described (29, 31, 32) and are thought to be associated with the respective levels of sex hormones. Our findings correspond with the majority of the literature; there were higher pro-inflammatory responses in females compared to males.

When the effects of smoking status and *IFNG* T + 874A genotype on IFN-γ levels were assessed, significant differences were observed between genotypes among smokers. In general, the effect of smoking was suppressive. The most dramatic effect was the 10-fold decrease in the response to the lower dose of endotoxin in smokers with the TT genotype compared to non-smokers. The only increase in IFN-γ responses in those who smoked was in individuals with the TA genotype when cells were stimulated with TSST-1. This was the most common genotype observed among SIDS infants from Hungary and Germany (Table 1).

The interaction of cigarette smoking with *IFNG* T + 874A genotypes on IFN-γ responses appears to be toxin specific. For example, for TSST, the TA genotype was greatly affected by cigarette smoke, while for endotoxin (0.01 μ ml^−1^) the TT genotype was most significantly affected by smoking. It is common to find conflicting data in the literature when assessing the function of a SNP, particularly when stimulation conditions differ. In our study, differences in responses were due to toxin type, as stimulation conditions were the same. We could not, however, control for asymptomatic infection or passive exposure to cigarette smoke as cotinine levels were not assessed in the studies with samples from SIDS parents and the unrelated comparison group.

There is little information in the literature on the effects of the *IFNG* SNP on cytokine responses. Two groups have found the TT genotype to be associated with increased IFN-γ production (22, 33, 34); however, smoking or environmental tobacco smoke (ETS) exposure had not been considered as a confounder. These results again highlight the need to control for smoking status and ETS exposure when assessing the effects of cytokine gene SNPs *in vitro*.

We have observed similar interactions with cytokine gene SNPs and smoking; IL-10 responses were significantly decreased in smokers with the *IL10* G-1082A AA genotype. IL-6 responses were significantly increased in smokers with the *IL6* G-174C GC (24, 25). Data from this study and previous findings suggest that exposure to cigarette smoke alters cytokine responses more for some genotypes than others.

There were significantly higher IFN-γ responses to TSST-1 from cells of donors with the TA genotype, and this was the predominant genotype among SIDS infants from Germany and Hungary (Table 1). Pyrogenic staphylococcal toxins have been detected in over 50% of SIDS infants from five different countries (20). Interactions between a mild virus infection and exposure to cigarette smoke in an infant with the TA genotype might lead to high levels of IFN-γ, which could result in significant down regulation of IL-10 and dysregulation of pro-inflammatory responses to pyrogenic toxins. In a vulnerable infant, this dysregulation of inflammation could trigger the physiological events leading to SIDS.

## Author Contributions

Each of the authors made substantial contributions to the conception, design, analyses, and interpretations of the work. They assisted in preparing the article, critically assessed the final version and agree to be accountable for the accuracy and integrity of the work.

## Conflict of Interest Statement

The authors declare that the research was conducted in the absence of any commercial or financial relationships that could be construed as a potential conflict of interest.
